# Utilization of biodiesel derived-glycerol for 1,3-PD and citric acid production

**DOI:** 10.1186/s12934-017-0807-5

**Published:** 2017-11-06

**Authors:** Laura Mitrea, Monica Trif, Adriana-Florinela Cătoi, Dan-Cristian Vodnar

**Affiliations:** 1 Department of Food Science, Faculty of Food Science and Technology, Institute of Life Sciences, University of Agricultural Sciences and Veterinary Medicine Cluj-Napoca, Calea Mănăştur 3-5, 400372 Cluj-Napoca, Romania; 2 Pathophysiology Department, “Iuliu Haţieganu” University of Medicine and Pharmacy, Cluj-Napoca, Romania

**Keywords:** Biodiesel, Crude glycerol, 1, 3-PD, Citric acid, Strains, Fermentation

## Abstract

Today, biofuels represent a hot topic in the context of petroleum and adjacent products decrease. As biofuels production increase, so does the production of their major byproduct, namely crude glycerol. The efficient usage of raw glycerol will concur to the biodiesel viability. As an inevitable waste of biodiesel manufacturing, glycerol is potentially an attractive substrate for the production of value-added products by fermentation processes, due to its large amounts, low cost and high degree of reduction. One of the most important usages of glycerol is its bioconversion through microbial fermentation to value-added materials like 1,3-propanediol and citric acid. There is a considerable industrial interest in 1,3-propanediol and citric acid production based on microbial fermentations, as it seems to be in competition with traditional technologies utilized for these products. In the present work, yields and concentrations of 1,3-propanediol and citric acid registered for different isolated strains are also described. Microbial bioconversion of glycerol represents a remarkable choice to add value to the biofuel production chain, allowing the biofuel industry to be more competitive. The current review presents certain ways for the bioconversion of crude glycerol into citric acid and 1,3-propanediol with high yields and concentrations achieved by using isolated microorganisms.

## Introduction and background

### Biodiesel

Due to the continuously growing of world industrial output, every quantity of energy is needed. This energy is provided through biological, chemical, electrochemical or physical ways and mechanisms, starting from natural resources. One of these natural resources is well-known as petroleum and its byproducts, like petrol, diesel, gasoline, etc. Due to the increased fuels demands on the market, these natural resources present some negative aspects because of the global ecological imbalance they have created. In this respect, an alternative fuel source is strongly necessary [1]. There are some researches which underline that petroleum production will decrease gradually until 2050, and the reserves are thought to become completely exhausted by then. Taking these into account, the demand for alternative fuels is growing worldwide and the use of biomass for producing biofuels is one of the most promising choices so far [2, 3].

**Scheme 1:**
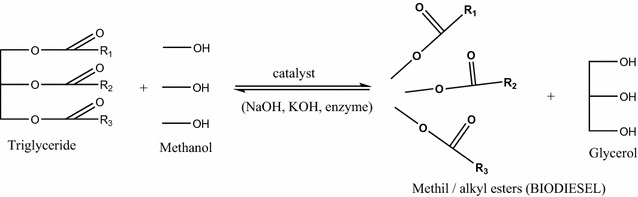
The general transesterification reaction of triglycerides in order to obtain biodiesel and glycerol

Biofuels represent a variety of combustibles which derive from biomass. In Europe, the best known biofuel is *biodiesel*. This particular type of fuel is created from animal fats, vegetable oils or recycled greases [4]. Biodiesel can be characterized as long chains of alkyl esters, which are formed by transesterification reaction (Scheme 1) of triglycerides with alcohol resulting in glycerol as a by-product [1]. During the biofuels manufacturing process, a great amount of residue is generated—in particularly *glycerol*—fact which leads to a negative aspect concerning the price of biodiesel. A general ratio between the biodiesel production and the amount of generated residual glycerol, points that for every 10 parts of biodiesel, one part of glycerol is produced [5–7].

### Crude-glycerol (CG), a byproduct of biodiesel production

Crude glycerol, in most cases, can be obtained in two ways: hydrolytically from oils and fats by soaps and fatty acids production, or by transesterification of fats or oils with an alcohol in the presence of a catalyst during the production of biodiesel. In the second method, the catalyst involved in the transesterification reaction may be an acid, a base, or an enzyme. Most often, base catalysts widely used are NaOH or KOH [6, 7]. After the transesterification reaction [3] and separation of crude biodiesel, crude glycerol is not pure enough for a direct use in different applications [8, 9]. In order to defeat this problem, impurities must be removed by an effective and very efficient purification process, to minimize the production costs and waste [8].

Biodiesel derived-glycerol also contains two substrates, namely glycerol and fatty acids. Hypothetical, these compounds can be used both simultaneously as well as gradually. In a study conducted by Morgunov and Kamzolova [9] it is presented that some specific strains are able to use both glycerol and fatty acid fractions during fermentation processes, even glycerol is consumed at a more elevated rate than fatty acids [9].

**Scheme 2:**
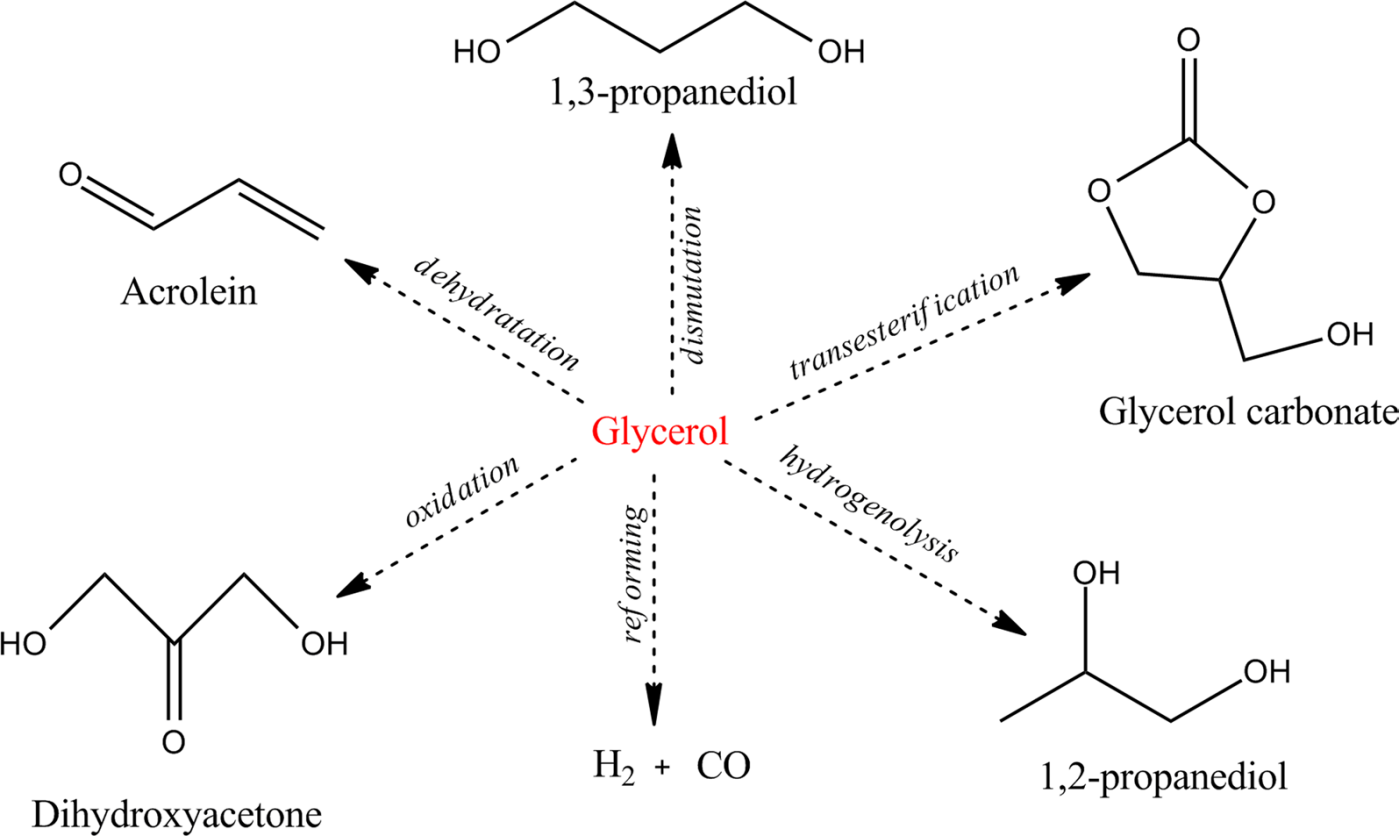
Possible pathways of glycerol degradation [14]; various products can be obtained

Glycerol, similar to multiple other small and uncharged molecules, can pass through the cytoplasmic membrane of different microorganisms. This passing occurs through passive diffusion. Many strains are able to develop on glycerol as a carbon source, due to the fact that this substrate can be both oxidatively and reductively metabolized through dehydrogenase or dehydratase. In this respect, by using yeast, bacterial and fungal strains, lots of value-added metabolic compounds could be obtained through microbial fermentation of glycerol, such as: acetic acid, lactic acid, propionic acid, citric acid, succinic acid, oxalic acid, butanol, propanediol, mannitol, ethanol, dihydroxyacetone, single-cell oil, biomass, polyunsaturated fatty acids, etc. (Scheme 2) [10–13].

The biodiesel production process implies a glycerin phase obtained as by-product. This phase contains glycerol, methanol, mono- and diaceloglycerols, fatty acids [4, 9], and soaps. Some statistics reveal that impressive quantities of glycerin phase are generated each year (at least 200–300 tons, up to hundreds of thousand tons per year), depending on the biodiesel production industry of each country. This fact leads to environmental problems regarding the management of this by-product [4, 10]. An appropriate solution to this threat is the usage of crude glycerol or glycerin phase as a carbon source for microbial growth media used in the production of various types of metabolites, for example 1,3-PD, citric acid, lactic acid, propionic acid, succinic acid and dihydroxyacetone [4, 11, 15]. The conversion of CG into value added products represents a strategy for the economic recycle of waste, and an approach for utilization of raw glycerol as a source for production of different industrial value-added products [1].

From the chemical point of view, pure glycerol is a liquid substance with no odor or color, which is hygroscopic and viscous with a vague sweet taste. Industrially obtained raw glycerol, is a light brown semi solid substance resulted as waste of biodiesel production [16]. Glycerol presents three hydrophilic alcoholic hydroxyl groups, which make it responsible for its good solubility in water and give it hygroscopic properties [7]. Glycerol density is 1.261 kg/L, it has a melting point of 18.2 °C, and a boiling point of 290 °C under pure anhydrous condition and normal atmospheric pressure [14]. In terms of ecological toxicity, the thermal degradation of glycerol at high levels of temperature (280–300 °C) can produce acrolein which is a poisonous compound for living organisms. That is to say, even a small quantity of acrolein (approximately 2 ppm) exerts strong toxicity [14]. In this context, the utilization of crude glycerol as a nutrient broth for bacteria appears as a viable future prospects [1]. Glycerol has many other applications in different areas; it is largely used as commodity chemical in pharmaceutical industry and in the production of dyes, cosmetics, soaps, toothpaste, lubricants, food, antifreeze solutions, etc. [6, 17]. The enormous amounts of glycerol resulted from manufacturing of biodiesel make the utilization of CG cheaper, as a carbon source, compared with glucose. More than that, glucose is directly implied in food production, while glycerol is not, which makes it a feasible carbon source for various fermentations [1, 14].

In the field of biochemistry, glycerol performs a fundamental role in the stabilization of enzymes due to its polyhydric alcohol functions. This fact generally enhances the structural stability of the entire protein, by maintaining the equilibrium of the hydrophilic–lipophilic profile (HLB) which is achieved by means of protein adsorption. It can be concluded that glycerol presents a major importance, as this compound also secures the biological compounds during sol–gel entrapment in matrices based on silica, by forming poly-glyceryl silicate as sol–gel precursors, or by addition in direct way to the microorganisms preceding the sol–gel poly-condensation [7].

## The bioconversion of glycerol to 1,3-propanediol (1,3-PD)

From the chemical point of view 1,3-propanediol is also named trimethylene glycol, 1,3-dihydroxypropane, or propane-1,3-diol. Its molecular formula is C_3_H_8_O_2_, and it has a molecular mass of 76.09 g × mol^−1^. 1,3-PD’s boiling point is 210–212 °C and the melting point is − 28 °C [4, 18]. 1,3-PD represents a specific product of glycerol fermentation, and it is a chemical intermediate largely used in the manufacture of polymers (polyethers, polyesters, polyurethanes), drugs, cosmetics, lubricants, and is also used as a mediator in the synthesis of heterocyclic compounds [3, 4, 19]. Recent studies reveal that 1,3-PD is often used as a monomer to synthesize a new type of biodegradable polyester, namely polytrimethylene terephthalate (PTT), which is more environmentally friendly and holds better properties then other plastic materials, like polyethylene terephthalate (PET) or polybutylene terephthalate (PBS) [6, 19, 20].

**Scheme 3:**
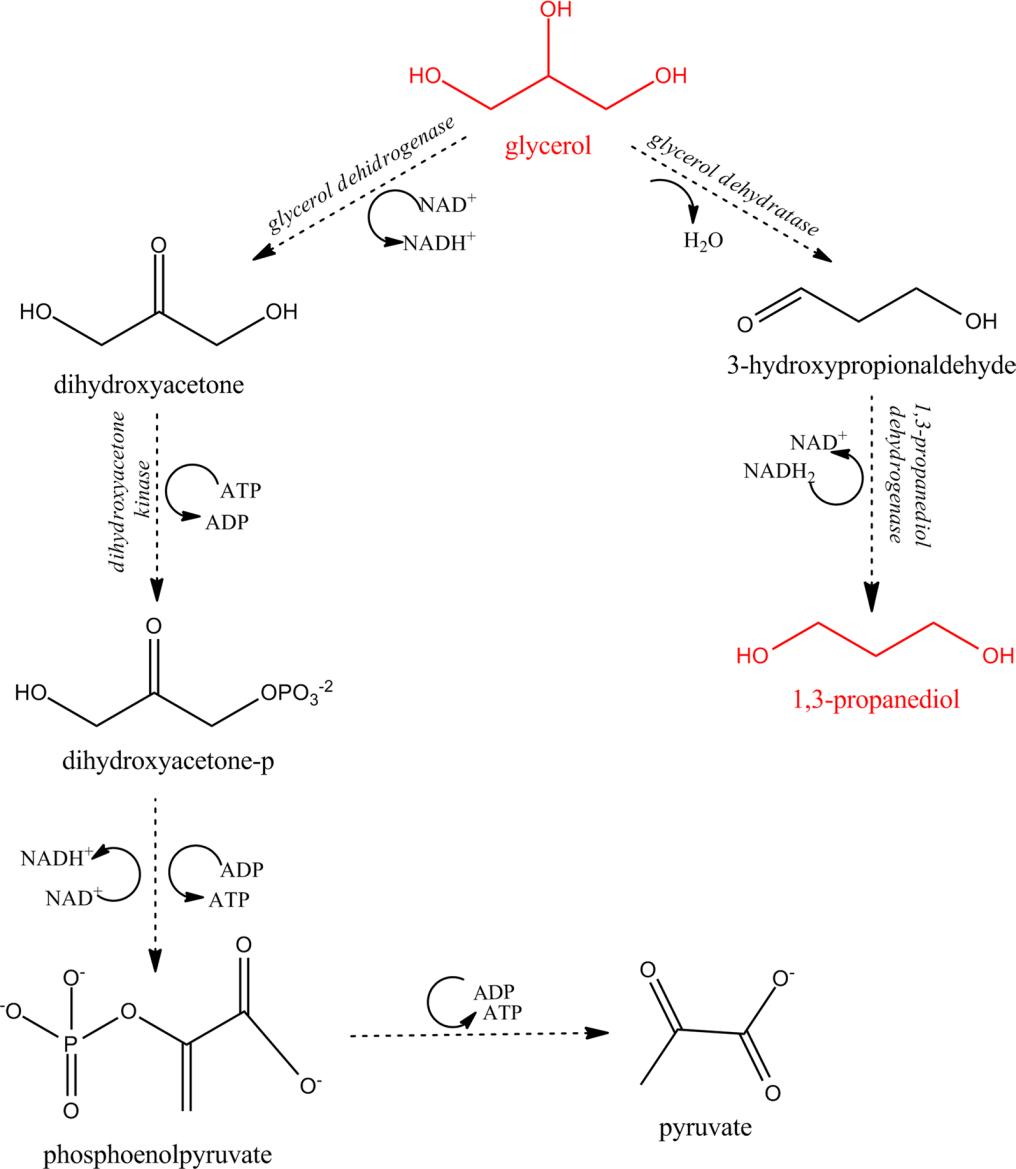
Collateral pathways of glycerol fermentation [4, 19, 23]; the mechanism of 1,3-PD production

From a metabolic point of view, glycerol is fermented through dismutation [2] which involves two collateral pathways. There is one pathway where crude or pure glycerol is transformed into dihydroxyacetone by a glycerol dehydrogenase, and there is another one where a coenzyme B12-dependent glycerol dehydratase transforms glycerol to 3-hydroxypropionaldehyde. In the last mentioned pathway, 3-hydroxypropionaldehyde is reduced to 1,3-PD by the 1,3-propanediol dehydrogenase NAD^+^ dependent enzyme (Scheme 3), under the consumption of reducing power (NADH_2_) [3]. The NADH_2_ generated through the glycerol metabolism leads to the formation of various byproducts using the important glycolysis reactions [21]. Moreover, the NADH_2_ supplementation and regeneration are critical in order to obtain great yields and concentrations of 1,3-PD [22]. In this way, many other metabolites can be obtained from glycerol, considering the two fermentation pathways (dihydroxyacetone, 2,3-butanediol, acetic acid, propionic acid, succinic acid, citric acid, lactic acid, docosahexanoic acid, hydrogen, ethanol) [1, 4, 6, 19, 21, 22].

The chemical synthesis of 1,3-propanediol can be conducted by two significant processes. The first one is ‘‘Degussa’’ (hold by ‘‘DuPont Company’’) and implies catalytically oxidation of propylene to acrolein, which is hydrated next to 3-hydroxypropionaldehyde at medium pressure and temperature, followed by the hydrogenation to 1,3-PD using a rubidium catalyst at high pressure. The second process carried out by ‘‘Shell’’ is based on oxidation of ethylene to ethylene oxide, followed by production of 3-hydroxypropionaldehyde through the reaction called ‘‘hydroformylation’’ (also named ‘‘oxo synthesis’’) at high pressures (around 150 bar). The aldehyde extraction from the organic phase is performed using water, and the 3-hydroxypropionaldehyde hydrogenation is conducted by using nickel as a catalyst under high pressure. The production of 1,3-PD registers a conversion yield between 65 and 80% when acrolein and ethylene oxide are used as raw material [21, 24].

For the quantification of kinetic behavior (like cell growth, substrate assimilation, final product synthesis) of able microorganisms to convert pure or crude glycerol directly to 1,3-PD, modeling approaches were used for different wild strains [25–28]. Some authors described the glycerol bioconversion to 1,3-PD by showing a series of chemical reactions along with some mathematical equations regarding the biomass yield [25, 26, 28], substrate consumption and 1,3-PD production rates [28]. In their studies, Papanikolaou and others [27, 28] simulated a modified Monod’s equation, namely the Contois-type model, capable to predict the production of biomass and 1,3-PD from glycerol by wild *Clostridium butyricum* F2b in fed-batch cultivations [27]. Using Contois-type model was found that the maximum theoretical productivity of 1,3-PD was comparable with the highest one obtained during growth of various bacterial strains cultivated on pure glycerol in batch and fed-batch cultures [27].

More and more, new approaches for natural production of 1,3-PD are deeply studied. The employment of microorganisms to produce 1,3-PD from crude glycerol represents one important topic for the research field. A generous number or microorganisms can develop anaerobically on glycerol, as nutrient and energy source. In present, several strains acting as biocatalysts are used and well investigated for 1,3-PD production. We can mention some examples of good 1,3-PD producers: *K. pneumoniae*, *K. oxytoca*, *K. planticola*, *C. freundii*, *Cl. butyricum*, *L. brevis*, *L. buchneri* etc. [6, 29].

### *KLEBSIELLA’s* 1,3-PD production

Among the strains of *Enterobacteriaceae*, *K. pneumoniae* seems to give the best results in 1,3-PD production. In order to obtain good results, Mu et al. [6] proposed an integrated bioprocess combining biodiesel production via lipase, with microbial production of 1,3-PD by *K. pneumoniae* DSM 2026, using a hollow fiber membrane [6]. During the process, the microorganism has converted glycerol directly to 1,3-PD, with the following results: the final concentration of 1,3-PD was about 61.1 g/L, the molar yield was 0.51 mol/mol, and the volumetric productivity of 1,3-PD was 2.0 g/L/h. There was also mentioned the maximum theoretical yield of 1,3-PD to glycerol, which was 0.72 mol/mol under anaerobic conditions [6].

Classical reports also show that wild *Klebsiella* strains are potential producers for relatively important values of 1,3-propanediol. A study conducted by Menzel et al. [26] in 1997 points that *K. pneumoniae* DSM 2026 produces about 35.2–48.5 g/L of 1,3-PD with theoretical maximum yield of 0.721 mol/mol and a volumetric productivity ranging between 4.9 and 8.8 g/L/h, in a continuous and anaerobic fermentation of glycerol [26].

Another research revealed that Rossi et al. [30] tested a consortium of bacteria in order to find a productive strain for 1,3-PD and ethanol from crude glycerol as a carbon source. Consequently, *Klebsiella pneumoniae* BLh^−1^ seems to give best results in degradation of CG. The consumption of CG was entirely performed within 32 h of cultivation in anaerobic conditions in a bioreactor, with a production of 1,3-PD of 19.9 g/L, and a theoretical yield of 0.72 mol product/mol glycerol. The same paper suggests that *K. pneumoniae* BLh^−1^ gives similar results on pure glycerol too, as carbon source, realizing a production of 22.8 g/L of 1,3-PD, with yields (Yp/s) of 0.68 mol product/mol glycerol [30].

From many points of view, *K. pneumoniae* is one of the most investigated and efficient microorganisms for 1,3-PD production from crude or pure glycerol. A newly isolated strain, namely *K. pneumoniae* GLC29 was intimately investigated by Da Silva et al. [31]. Beside glycerol concentration, the effects of some parameters as pH, temperature and stirrer speed on the production and productivity of 1,3-propanediol were also evaluated. Considering both production and productivity, the best conditions for conversion of glycerol in 1,3-propanediol are as follows: a pH range of 6.9–7.1, a temperature between 33 and 38.5 °C, a stirrer speed range of 110–180 rpm, and a glycerol concentration of 39–49 g/L. The batch fermentation performed at a pH of 7.0, a temperature of 35°C, a stirrer speed of 150 rpm, and a glycerol concentration of 40 g/L produced 20.4 g/L of 1,3-PD, with a maximal volumetric productivity of 2.92 g/L/h and a yield of 0.51 g/g. Few byproducts were obtained, like acetic acid (approximately 7.0 g/L) and formate (approximately 3.7 g/L). It can be concluded that the novel *K. pneumoniae* GLC29 showed potential for the conversion of glycerol into 1,3-propanediol, with high production yields and productivity [31].

Strains as *Klebsiella oxytoca* were reported by Garlapati et al. [1] to transform crude-glycerol into 1,3-PD under batch and fed-batch fermentation conditions with a yield and productivity ranging from 0.41 to 0.53 g/mol, respectively from 0.63 to 0.83 g/L/h [1].

### *CLOSTRIDIUM’s* 1,3-PD production

Few studies regarding the bioconversion of CG into 1,3-PD using strains from *Clostridium* genus, have been reported. Some researches reveal that *Clostridium butyricum* F2b leads to good results [32], and produce important amounts of 1,3-propanediol on crude glycerol as a carbon source, in a continuous mode. Papanikolaou and others [32] grew *C. butyricum* F2b microorganisms on crude glycerol used as sole substrate, at concentrations of 39 and 90 g/L. The biomass production observed ranged between 1.2 and 2.6 g/L, while the productivity of 1,3-PD reached a maximum concentration of 47.1 g/L, and was the principal metabolic product [32]. Papanikolaou et al. [28] suggest that raw glycerol is a suitable source for the development of *C. butyricum* F2b and 1,3-PD production, in batch and single-stage continuous cultures. They underline that high intake of substrate concentrations positively influence the synthesis of 1,3-PD, favoring the production of organic acids like acetic acid and butyric acid. This fact is considered to be due to the organic acids metabolic pathway, which is a competitive and alternative pathway to that of 1,3-PD in the microbial cell [28].

A spore forming and anaerobic wild-strain, namely *Clostridium* species IK124, was tested by Hirchmann et al. [33] to evaluate its potential of using the untreated glycerol from biodiesel industry as a main substrate for 1,3-PD production. During fed-batch fermentation they combined the low base-driven glycerol addition with the constant glycerol measurement and a feedback regulation. Using this strategy and the *Clostridium* IK124 the results were significantly high (final 1,3-PD concentration: 87 g/L; productivity: 2,2 g/L/h; yield: 65% [mol/mol]) [33].

*Clostridium butyricum* strain VPI 1718 was investigated by Chatzifragkou et al. [34] in order to evaluate the production of 1,3-PD on crude glycerol as carbon source, and the impact of various impurities that can be found in CG derived from biodiesel production. The preliminary trials in 200 mL anaerobic flasks revealed that the presence of salts might influence the cell growth. Salts like NaCl (4.5% w/w of glycerol) imposed an evident inhibitory effect in the growth medium, while phosphoric salts did not. Anyhow, NaCl appeared to show no influence on large quantities pending batch bioreactor experiments [up to 30% (w/w of glycerol)], and the microbial growth and 1,3-PD production are not affected by this compound. In this respect, *C. butyricum* VPI 1718 possesses significant tolerance capacity against specific salt quantities found in crude glycerol. Moreover, the presence of methanol did not influence the bacterial bioconversion of glycerol to 1,3-PD, even when relatively high concentrations(10%, w/w, of glycerol) were imposed in batch-reactor fermentations. Methanol was added, during continuous experiments, when steady state had been accomplished. Even though a high concentration of methanol was added into the fermenter (5 g/L), the system gained a steady state without indicating any of the negative effects over biomass production due to the presence of alcohol [34]. By comparison of the biochemical response of the bacteria during utilization of pure or crude glycerol, it can be noticed that crude glycerol had no conspicuous effect on *C. butyricum* VPI 1718 in respect to both microbial growth and 1,3-PD production. Specifically, by the time of the continuous operation, 1,3-PD production recorded 14.1 g/L, reaching a volumetric productivity of 1.41 g/L/h. At this dilution rate, important glycerol uptake was noticed, yielding a value of 1.08 g/g/h [34].

The same strain, *C. butyricum* VPI 1718, was tested [35] during a fed-batch operation under non-sterile fermentation conditions. Crude glycerol was employed as a nutrient source. The final concentration of 1,3-PD was 69.7 g/L, with an yield of 0.55 g/g, and a maximum volumetric productivity of 1.87 g/L/h [35]. More than that, the bioreactor’s geometry and the effect of anaerobiosis strategy over the biochemical response of *C. butyricum* VPI 1718 were analyzed during 1,3-PD production [36]. It seems that the strain VPI 1718 can successfully produce 1,3-PD in the presence of N_2_ gas infusion whatever the initial glycerol concentration and bioreactor size are imposed [36].

Wilkens et al. [37] achieved good results regarding the biodiesel derived glycerol conversion to 1,3-propanediol, with *Clostridium butyricum* AKR102a. In a fed-batch fermentation, under anaerobic conditions, they obtained 93.7 g/L of 1,3-PD with an yield of 0.632 mol/mol, and an overall productivity of 3.3 g/L/h by using pure glycerol as the nutrient source. Using crude glycerol as the substrate under the same conditions, 76.2 g/L of 1,3-PD was produced with a yield of 0.622 mol/mol, and a productivity of 2.3 g/L/h [37].

Xin et al. [20] combined two sources of nutrient substrates in order to enhance the productivity of 1,3-PD. Therefore, they used lignocellulosic hydrolysates (glucose, xylose, and arabinose) in an anaerobic fermentation, as co-substrates for the increasing yield of glycerol conversion to 1,3-PD. The three mentioned sugars were used, separately but concomitantly with glycerol, in the production of 1,3-PD by a *Clostridium diolis* DSM 15410. The results were situated between 18 and 28%, meaning an increase in the 1,3-PD yield [20]. Beside the fact that glycerol is used as the sole carbon source in different fermentation processes, an addition of low-cost raw materials as co-nutrients may decrease the expenses of 1,3-PD production costs. There can be mentioned that glycerol is transformed in different metabolites during the dismutation process, via reductive and oxidative branches. The first one (reductive branch) determines 1,3-PD production with the consumption of NADH, and the glycerol is oxidized to metabolites such as H_2_, CO_2_, acetate, butyrate, lactate, ethanol, butanol, or 2,3-butanediol. On this pathway energy is produced and the cell growth is reduced, facts which lead to the decrease of 1,3-PD production. Moreover, when glycerol is utilized as the sole nutrient substrate, the conversion yield of glycerol to 1,3-PD ranges usually under 0.72 mol/mol (the theoretical yield) [20]. Xin et al. [20] point that the co-utilization of glycerol and glucose as carbon sources increases the cell growth, and at the same time, the production of 1,3-PD, which reaches to 14.7 g/L. The yield of 1,3-PD to glycerol when glucose, xylose, and arabinose were co-utilized with glycerol, increased by 28% (0.86 mol/mol), 19% (0.80 mol/mol), respectively 18% (0.79 mol/mol). Therefore, lignocellulosic hydrolysates such as glucose, xylose, and arabinose could be considered as supplement resources in glycerol fermentation in order to increase the 1,3-PD production yields [20].

Nakas et al. [38] obtained 5 g/L of 1,3-PD besides ethanol and butanol from 49 g/L glycerol using the strain of *Clostridium pasteurianum* [38].

### *CITROBACTER’s* 1,3-PD production

*Citrobacter freundii* seems to be another promising organism for 1,3-PD production among *Enterobacteriaceae.* Casali et al. [39] compared the 1,3-PD producing potential of *Citrobacter freundii* strain DSM 15979, with *Pantoea agglomerans* DSM 30077, from crude glycerol as carbon source. The optimal quantity of raw glycerol which gave the highest 1,3-PD productivity, was about 40 g/L at an average concentration of 20–60 g/L used in preliminary studies. The final 1,3-PD concentration obtained using *C. freundii* was 12.92 g/L, while 6.14 g/L was obtained for *P. agglomerans*. Both mentioned strains were able to accrue on crude glycerol leading to an accumulation of 1,3-PD in the cultural broths. From this report it can be observed that even if *P. agglomerans* is a novel bacterium in the field of CG conversion to 1,3-PD and it is not well investigated yet, it appears as a promising strain with appropriate yields to the 1,3-PD production [39].

Boenigk et al. [40] studied the process of glycerol conversion to 1,3-propanediol by *Citrobacter freundii* DSM 30040. The process was optimized in single- and two-stage continuous cultures. The production of 1,3-PD was increased under glycerol limitation and elevated with the dilution rate (D) of 3.7 g/L/h. The optimal conditions for the two-stage fermentation process were as follows: (a) first stage—glycerol limitation at 250 mM, pH 7.2, D = 0.1 h^−1^, 31 °C; (b) second stage—additional glycerol, pH 6.6, D = 0.05 h^−1^, 28° C. In these terms, the final concentration of 1,3-PD was 545 mM, and the concentration of consumed glycerol were 875 mM. The average productivity of 1,3-PD recorded 1.38 g/L/h. In order to gain a continuous productivity of 1,3-PD by conversion of glycerol, Boenigk et al. [40] mentioned that a growth limitation by nitrogen source or by phosphate could be helpful. This might enable glycerol to be present excessively in the medium and achieve maximum values of 1,3-PD concentrations. Taking into account these growth limitations, 2.9 mM of ammonium or 0.75 mM of phosphate in a medium culture supplemented with 0.02% yeast extract, *C. freundii* DSM 30040 grew to an optical density (OD_578_) of 1.3. In contraposition to batch cultures, cells were extended and occurred in chains. In this case, cells were not highly productive in formation of 1,3-propanediol, and the specific activities of the responsible enzymes, like glycerol dehydratase and 1,3-propanediol dehydrogenase, were very low (data not shown) [40].

Metsoviti et al. [41] investigated the isolated *Citrobacter freundii* strain FMCC-B 294 (VK-19) for its potential of converting the biodiesel-derived glycerol into 1,3-propanediol. Their study demonstrated that raw glycerol used as a nutrient substrate was very effective for both *C. freundii* growth and 1,3-PD production. At the same time, their study proved that batch fermentations conducted in non-sterile conditions do not influence considerably the final concentration of 1,3-propanediol. In this regard, the research group obtained 68.1 g/L of 1,3-PD with an yield of consumed glycerol of 0.40 g/g and a volumetric productivity of 0.79 g/L/h during a sterile fed-batch fermentation, while 66.3 g/L of 1,3-PD were obtained from 176 g/L of raw glycerol, performing non-sterilized fed-batch process. From this research it can be concluded that *Citrobacter freundii* strain FMCC-B 294 can grow and can convert efficiently biodiesel derived-glycerol into 1,3-propanediol in non-sterile conditions [41].

### Novel strains and mutants

New strains have been modified in order to obtain a higher production of 1,3-propanediol. For example, Hartlep et al. [42] obtained glycerol using yeast named *Pichia farinosa* or an *E. coli* genetically modified strain, whereupon glycerol was converted to 1,3-PD by *K. pneumoniae*, and the overall yield was about 0.17 g/g [42]. Further advance has been performed by DuPont and Genencor Company. They employed a genetically modified bacteria using enzymes from strains as *Saccharomyces* and *Klebsiella* combined in one strain of *E. coli* K12, which transforms glucose directly to 1,3-PD with a final concentration of 130 g/L, but only with a low yield of 0.34 mol/mol [43].

Even the utilization of crude glycerol during fermentation process gives some advantages in relation to the use of pure glycerol, there are few reports on the potential use of crude glycerol as a biodiesel by-product for the production of 1,3-propanediol. Most of them are conducted using pure glycerol as a sole carbon source [30].

In terms of 1,3-PD production, a novel strain has been investigated, namely *Shimwellia blattae* ATCC 33430. In a 2 L bioreactor, Rodriguez et al. [44] tested different concentrations of raw glycerol (between 20 and 70 g/L), during 6 experiments repeated three times. The best results were obtained for 29.5 g/L of CG, representing a 1,3-PD concentration of 13.6 g/L, a yield of 0.49 g/g and a productivity rate of 1.36 g/L/h. The metabolic carbon flux switched to the oxidative pathway (to lactic acid and ethanol synthesis) when the initial concentration of glycerol was more elevated than 47.4 g/L [44].

Strains from genus *Lactobacillus* have been found to produce significant amounts of 1,3-PD. Pflügl et al. [45] evaluated the capability of *Lactobacillus diolivorans* DSM 14421 to develop on glycerol as a nutrient source and to produce 1,3-propanediol. Concentrations of 41.7 g/L for 1,3-PD were obtained in batch cultivation, while 73.7 g/L were obtained in fed-batch cultivation when glycerol was co-fermented with glucose. Same authors suggest that vitamin B12 added as supplement to the culture medium has increased the production of 1,3-PD to a final concentration of 84.5 g/L [45].

In Table 1 are mentioned several strains producing 1,3-PD using crude or pure glycerol as sole carbon source.

**Table 1:** The concentrations and yields of 1,3-PD obtained from crude and pure glycerol by various strains

Strain	Carbon source	Fermentation type	1,3-PD concentration (g/L)	1,3-PD yield (mol/mol)	1,3-PD productivity (g/L/h)	References
*Klebsiella pneumoniae* DSM 2026	Crude glycerol	Combined bioprocess	61.1	0.51	2.0	[6]
*Klebsiella pneumoniae* DSM 2026	Pure glycerol	Batch, fed-batch	35.2–48.5	0.721	4.9–8.8	[26]
*Klebsiella pneumoniae* BLh^−1^	Crude glycerol	Batch	19.9	0.72	–	[30]
*Klebsiella pneumoniae* GLC29	Pure glycerol	Batch	20.4	0.51	2.92	[31]
*Klebsiella oxytoca*	Crude glycerol	Batch, fed-batch	–	0.41–0.53	0.63–0.83	[1]
*Clostridium butyricum* F2b	Crude glycerol	Batch	47.1	–	–	[32]
*Clostridium* IK124	Crude glycerol	Fed-batch	87	65	2.2	[33]
*Clostridium butyricum* VPI 1718	Crude/pure glycerol	Batch	14.1	1.08	1.41	[34]
*Clostridium butyricum* VPI 1718	Crude glycerol	Fed-batch	69.7	0.55	1.87	[35]
*Clostridium butyricum* AKR102a	Crude/pure glycerol	Fed-batch	76.2/93.7	0.622/0.632	2.3/3.3	[37]
*Clostridium diolis* DSM 15410	Pure glycerol	Batch	14.7	0.86	1.1	[20]
*Clostridium pasteurianum*	Crude glycerol	Batch	5	–	–	[38]
*Citrobacter freundii* DSM 15979	Crude glycerol	Batch	12.92	–	–	[39]
*Citrobacter freundii* DSM 30040	Pure glycerol	Batch	60	–	1.38	[40]
*Citrobacter freundii* FMCC-B 294	Crude glycerol	Batch	68.1	0.4	0.79	[41]
*Pantoea agglomerans* DSM 30077	Crude glycerol	Batch	6.14	–	–	[39]
*E. coli* K12	Pure glycerol	Fed-batch	130	0.53	2.0	[43]
*Lactobacillus diolivorans* DSM 14421	Pure glycerol	Fed-batch	85.4	0.57	0.85	[45]
*Shimwellia blattae* ATCC 33430	Crude glycerol	Batch	13.6	0.49 g/g	1.36	[44]

## 1,3-PD purification

Considering the fact that 1,3-propanediol is a monomer obtained through fermentation process in passably low quantities, there is a demand for purification in order to gain a clear final product. Therefore, a series of separation techniques have been developed. According to the literature, there are three main steps in the purification process of 1,3-PD, and these are as follows: first of all, there is a removal of biomass (proteins and cells) through flocculation, membrane filtration, and high-speed centrifugation; second, there is a concentration of 1,3-PD through extraction, electrodialysis, and absorption; and third, there is a refining of high-purity 1,3-PD through vacuum distillation or distillation under reduced pressure [46].

Several studies reveal that there exist other methods for 1,3-PD purification. For example, Li et al. [47] reported that they recovered more than 95% of 1,3-PD by using an aqueous two-phase system for extraction. Anyway, this procedure seems not to give a high purity of the end product [46]. Anggraini et al. [48] separated the fermentation products from other compounds beside 1,3-PD by using the atmospheric distillation method, and they evaluated the purity of investigated product using The Gas Chromatography system (GC) [48]. High performance liquid chromatography (HPLC) is often used for concentration evaluation of separated products.

A successful commercialization of 1,3-propanediol from biological sources imposes an improvement of an efficient purification system. In these terms, a fermentation broth which contains a plurality of components, like residual glycerol, water, glucose, by-products (acetate, lactate, succinate, ethanol and 2,3-butanediol), macromolecules (proteins, polysaccharides and nucleic acid), salts and residual medium, makes the 1,3-PD downstream process separation quite difficult [49].

Methods of 1,3-PD purification have been analyzed by many researchers in previous studies. There can be mentioned a few examples of important procedures for 1,3-PD recovery: reactive extraction, liquid–liquid extraction, evaporation, distillation, membrane filtration, pervaporation and ion exchange chromatography. In this regard, all these methods have some drawbacks or limitations [49, 50]. In the context of reactive extraction, Broekhuis et al. [51] attempted to convert the targeted product into a compound without hydroxyl groups and then recovered it through solvent extraction. They used formaldehyde or acetaldehyde in order to create dioxane derivatives of 1,3-propanediol and 1,2-propanediol [47, 51].

Malinowski [52] studied liquid–liquid extraction, a method which can be used straight to recover a targeted product from a dilute solution, in case that a proper solvent is found. In his study, with the aid of extraction screening program (ESP), Malinowski performed a solvent screening where aldehydes and aliphatic alcohols were selected. The fact that 1,3-PD distribution into extraction solvents showed relevant differences between the theoretical and experimental values, made the whole process unsatisfactory in developing a simple and efficient extraction procedure [52].

The conventional techniques such as evaporation and distillation used for the removal of water and for 1,3-PD purification, consume high energy and lead to a raised price of the targeted product [49]. In the case of the vacuum distillation based separation process, it can be mentioned that it consumes less energy, due to the decrease of the boiling points. Still, this technique used for the recovery and purification of 1,3-PD gives low yields because it makes broth very viscous, and leads to low evaporation efficiency [49].

Cation exchange resin of polystyrene sulfonate in the Na form was used by Hilaly and Binder [53] for a strong separation of 1,3-propanediol from other compounds. With the aid of a simulated moving bed apparatus and added water as an eluent for the feed material, the process was performed. In this regard, the original feed solution was diluted by ten times. The results were satisfactory, recording a yield of 1,3-PD higher than 95%. Even though high purity and yields of 1,3-PD can be obtained, the selectivity and capacity of the resin seem to be very low, so the 1,3-PD solution must be diluted, not concentrated. This method consumes a lot of energy compared to the simple evaporation and distillation methods. Moreover, the chromatographic matrix had to be regenerated frequently due to the fact that the feed was not desalinated or deproteinized [49, 53].

The purification of 1,3-propanediol by using an ion exchange comprising a strong acidic cation exchange resin and a weak basic anion exchange resin, was used in the removal of anionic and cationic molecules by Adkesson et al. [54]. This process implies the ion exchange resin regeneration more frequently, because of great amounts of anionic and cationic molecules in fermentative broths [49, 54].

Wilkins and Lowe [55] tested a chromatography process in order to remove 1,3-propanediol, aiming to prevent the feedback inhibition of cell growth and product formation in the fermentation process [49, 55]. Also, a liquid chromatography column packed with silica resin was evaluated for the separation of 1,3-PD from a 1,3-propanediol and 1,2-propanediol mixture after phase separation using ethyl acetate [49, 56].

Regarding the 1,3-PD purification, Saxena et al. [49] would particularly mention a novel technique based on their recent studies on purification of 1,3-PD from the fermentation broth. They separated the 1,3-PD in three main steps: proteins ejection, broth concentration, and 1,3-PD separation through chromatography. The mentioned method assumes relatively simple equipment and an easy maintenance compared to other available techniques. In Saxena’s study, a cheaply available compound was used as compared to the expensive chitosan for protein removal, thereby reducing the cost of the procedure [49]. The broth concentration was achieved by the vacuum distillation method, and the concentrated broth was purified afterwards by chromatography. In their study Saxena et al. [49] proved that 1,3-PD can be recovered from the fermentation broth with this technique, which resulted in a yield of 98%. It can be mentioned that their process is simple, efficient and fast, and it avoids the high price obstruction caused in the commercialization of 1,3-PD production [49].

## The bioconversion of glycerol to citric acid (CA)

**Scheme 4:**
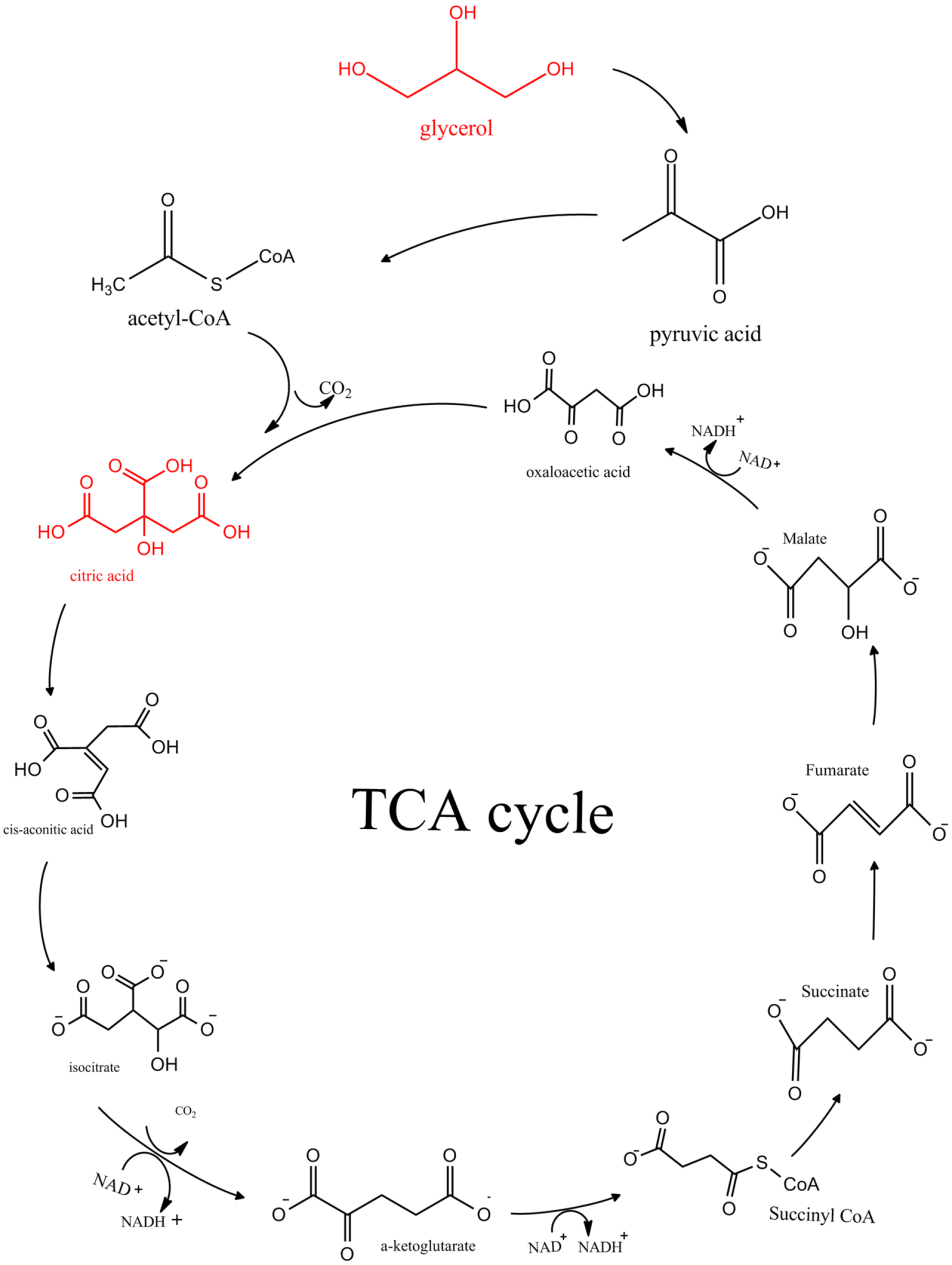
General pathway of citric acid production [60, 62–64]

Citric acid is well-known industrial product and can be obtained primarily by fermentation. Because of its low toxicity compared with other acidulants, the citric acid is used to add a pleasant and astringent flavor to beverages and aliments [57]. In terms of biochemical properties, the citric acid, also named 2-hydroxy-propane-1,2,3-tricarboxylic acid, was first described as a compound obtained from citrus plants and known as an intermediate of the tricarboxylic acid (TCA) cycle (Scheme 4). CA combines an enjoyable taste with an impressive palatability, so it becomes a ubiquitous food additive [58]. CA is also applied as an additive in functional detergents, in pharmaceuticals, and cosmetics. With a global annual production over 1.7 million tons, CA is noticed to be the first among the organic acids synthesized by bacteria. Multiple microorganisms, mainly *Yarrowia lipolytica* and *Aspergillus niger* are used to ferment various nutrient sources in order to obtain high yields of CA. As an example, *Y. lipolytica* can grow on a variety of carbon sources, like sugars, plant oils, alkanes, hydrolysates, ethanol, and glycerol under nutrient-limited conditions, and is competent to produce CA [57, 59–61].

The chemical conversion of glycerol can be interpreted in three manners: (a) by oxidation and reduction of glycerol into other 3-carbon compounds; (b) by synthesis of higher carbon compounds with glycerol and other substrates; (c) by industrial combustion [23]. These are traditional chemical catalytic methods, and often involve expensive metal catalysts, toxic intermediate compounds, and low conversion rates. Furthermore, it is very difficult to burn glycerol productively because of its high viscosity, low energy density, high auto-ignition temperature, and potential emission problems. In this context, glycerol conversion using microorganisms is a viable option compared to the direct application and chemical transformation, and in this way, certain drawbacks such as low product specificity, intensive pretreatment requirements, and high energy intake (pressure/temperature), can be avoided [23].

The industrial citric acid production can be carried also, in three different ways: by submerged fermentation, surface fermentation and solid-state fermentation [57]. All of the mentioned methods demand excellent raw material and a proper production fermenter for a direct strain inoculation. Soccol et al. [57] underline that CA accumulation is strongly influenced by the type and the concentration of the carbon source [57]. The carbohydrates are rapidly consumed by microorganisms and they are essential for a better production of citric acid. As examples of the more easily metabolized carbohydrates, sucrose is the most favored carbon source, followed by glucose, fructose and galactose [57].

During the glycerol fermentation by specific strains, citric acid synthesis implies depletion of nitrogen source from the cultivation media [32, 64]. The nitrogen exhaustion leads to a sudden decrease of intracellular AMP concentration, due to its separation caused by AMP-desaminase. Thereby, NAPD^+^—isocitrate dehydrogenase (the enzyme which is responsible for iso-citric change to a-ketoglutaric acid) loses its activity due to the fact that it is allosterically activated by the intracellular AMP, phenomenon which leads to the CA accumulation within the mitochondria [64]. If the citric acid concentration exceeds the critical value, CA is secreted into the cytoplasmic matrix. There are some oleaginous yeasts which are able to dissociate the cytoplasmic CA into acetyl-CoA and oxaloacetate by using ATP-citrate lyase (ACL), an enzyme responsible for lipid accumulation process. Acetyl-CoA is subjected next to a quasi-reversed β-oxidation reaction to cellular fatty acids [65]. That is to say, citric acid is used by oleaginous microorganisms as an acetyl-CoA donor in the anabolic pathway of fatty acids synthesis [64]. On the contrary, non-oleaginous microorganisms excrete the accumulated citric acid into the culture broth [63, 64].

Several synthetic routes have been developed by using different starting materials, but it is mentioned that chemical ways have been proved to be uncompetitive with the fermentation processes, mainly because the starting materials are much more expensive than the final product [62, 66].

### *YARROWIA’s* citric acid production

The increased yield of organic acid (citric acid) production by using crude glycerol has primarily been reported in strains of the *Yarrowia lipolytica* yeast. This microorganism was considered to be the most productive strain for citric acid converted from crude glycerol [8, 32, 60]. The cultivation of natural strains of *Y. lipolytica* on both crude glycerol from biodiesel production, and pure glycerol gave similar results regarding the CA yields. *Y. lipolytica* poses the ability to produce other valuable compounds, like biosurfactants, by fermentation of crude glycerol as sole carbon source [8].

In biotechnological processes, the biosynthesis of citric acid is performed in batch, fed-batch, as well as in repeated-batch cultures, occasionally with cell recycle and medium replacement. In their study, Rywińska et al. [60] investigated the yield and CA production of *Y. lipolytica* Wratislavia AWG7, which is an acetate-negative mutant with a smooth colony phenotype. The process was conducted under steady-state conditions, where the rising of the dilution rate was simultaneously performed by the reduction of CA concentration ranging from 86.5 to 51.2 g/L. Similar increase was recorded for the volumetric rate (from 0.78 to 1.59 g/L/h) and the specific rate (from 0.05 to 0.18 g/g/h) of citric acid production. The production process yield varied from 0.59 to 0.67 g/g [60].

In another research conducted by Rywińska et al. [67], one wild strain (*Y. lipolytica* A-101) was compared with three acetate-negative mutants of *Y. lipolytica* (Wratislavia 1.31, Wratislavia AWG7, and Wratislavia K1) regarding their capacity to produce CA from glucose and pure or raw glycerol in batch fermentations. The carbon source was used both as a single substrate and as mixtures of glucose and pure or raw biodiesel-derived glycerol. The final results pointed that the highest amount of CA were produced by A-101 (concentration: 82.2 g/L; yield: 0.45–0.52 g/g; productivity: 0.71–0.83 g/L/h), Wratislavia AWG7 (76.6 g/L; 0.46-0.48 g/g; 0.76–0.81 g/L/h) and Wratislavia 1.31 (71 g/L; 0.45–0.46 g/g; 0.57–0.69 g/L/h) strains from media containing a mixture of raw glycerol and glucose. On the other hand, Wratislavia K1 strain produced good quantities of erythritol (from 18.1 to 30 g/L) throughout the entire cultivation process, and lower quantities of citric acid (concentration: 36.8–53.3 g/L; yield: 0.27–0.3 g/g; productivity: 0.51–0.64 g/L/h) [67].

Strains like *Y. lipolytica* Wratislavia 1.31 and *Y. lipolytica* Wratislavia AWG7 were tested in fed-batch systems using different elevated concentrations of raw glycerol (200 and 300 g/L) as substrate for producing citric acid. For Wratislavia 1.31 strain, when 200 g/L of substrate were used, 126 g/L of citric acid were obtained within 120 h of fermentation, recording a yield of 0.63 g/g and a productivity of 1.05 g/L/h. For Wratislavia AWG7, when 200 g/L of substrate were used, 113.5 g/L of citric acid were produced within 121 h of fermentation (yield: 0.57 g/g; productivity: 0.94 g/L/h). When the total glycerol concentration was raised up to 300 g/L, with Wratislavia 1.31 strain it were achieved 155.2 g/L of citric acid (yield: 0.58 g/g; productivity: 0.6 g/L/h) and 157.5 g/L with Wratislavia AWG7 (yield: 0.55 g/g; productivity: 0.6 g/L/h) [68].

Rymowicz et al. [69] performed a batch fermentation in order to evaluate and compare the CA production of the named acetate-negative mutants of *Y. lipolytica* (K-1, AWG-7, 1.31). The principal source of carbon was crude glycerol (with an initial concentration about 200 g/dm^3^) obtained from biodiesel manufacturing, where rapeseed oil was used as raw material. At the end of the process, *Y. lipolytica* strain 1.31 gave best CA productivity, namely 124.5 g/dm^3^ of citric acid, with a yield of 0.62 g/g. Moderate values were registered for both *Y. lipolytica* K-1 and *Y. lipolytica* AWG-7 (75.7 g/dm^3^, y = 0.40 g/g for K-1 strain, and 88.1 g/dm^3^, y = 0.46 g/g for AWG-7) [69].

Another study conducted in 2010 by Rymowicz et al. [70] presents the CA producing potential of *Yarrowia lipolytica* A-101-1.22. This strain produced citric acid in concentration of 112 g/L, with a yield of 0.6 g of CA per g of consumed glycerol, and a productivity of 0.71 g/L/h during batch fermentation by using raw glycerol [70].

Rywińska and Rymowicz [71] obtained similar results (154 g/L of citric acid; a yield of 0.78 g/g and a productivity of 1.05 g/L/h) with *Y. lipolytica* Wratislavia AWG-7 when raw glycerol was fed in long-term repeated-batch cultures. They also investigated the culture activity which remained stable for more than 1650 h (16 cycles of the repeated-batch bioreactors) [71].

Kamzolova et al. [72] examined the potential of acids formation of 66 yeast strains from different genera, like *Candida, Pichia, Saccharomyces, Torulopsis* and *Yarrowia.* Among them, the mutant strain of *Yarrowia lipolytica* N15 was selected due to its ability to produce citric acid in high amounts. Under optimal conditions, the mutant *Y. lipolytica* N15 produced up to 98 g/L of CA (yield: 0.70 g/g; productivity: 1.14 g/L/h) when it is grown on medium containing pure glycerol, and 71 g/L of CA (yield: 0.9 g/g; productivity: 0.89 g/L/h) when it is grown on medium with glycerol-containing waste from biodiesel industry [72].

*Yarrowia lipolytica* strain NG40/UV7 was found to produce 115 g/L of citric acid in fed-batch fermentation when pure glycerol was added in the medium from 20 to 80 g/L. During the fermentation process containing raw glycerol (under the same conditions), 112 g/L of citric acid were produced [73]. In another trial, the mutant strain NG40/UV7 was tested during 192 h of fermentation when the culture media presented 20 g/L of glycerol-containing waste and 4 g/L of fatty acids [9]. The final concentration of synthesized citric acid was 122.2 g/L with a yield of 0.95 g/g and a productivity of 0.99 g/L/h [9].

Among the yeast strains, *Yarrowia lipolytica* NRRL YB-423 is shown by Levinson et al. [61] to produce the highest yield (54%) of citric acid (21.6 g/L CA from 40 g/L glycerol) [61]. These values were obtained by strain cultivation on pure glycerol. Also, crude glycerol was tested for citric acid production by *Y. lipolytica* NRRL YB-423, and the CA yield obtained with this substrate was 55.7% at the time of harvesting, and 94 mg/L/h was the rate of production over a period of 10-days of incubation. In this context, the yield and the production rate with crude glycerol are comparable to data obtained with pure glycerol used as a nutrient substrate. There have been reported similar CA yields based on raw glycerol resulted from a biodiesel production for *Y. lipolytica* ACA-DC 50109 strain [32, 61].

Papanikolaou and colleagues suggest that *Yarrowia lipolytica* strain ACADC 50109 [LGAM S(7)1] presented a moderate accumulation of citric acid in the medium reaction when they were cultivated on crude glycerol as substrate in nitrogen limited flask cultures [32]. Citric acid was produced after the exhaustion of nitrogen from the medium, resulting in a final quantity of 62.5 g/L and the yield on glycerol consumed was 0.56 g/g [32]. The very same strain *Y. lipolytica* ACADC 50109 produced around 50–55 g/L of citric acid on glucose in mono- or in dual substrate (productivity: 0.6 g/L/h in both cultures), under optimized conditions with pO2 control [74]. During the same trial, when glycerol was used in mono-substrate culture, 18 g/L of citric acid was obtained with a productivity of 0.2 g/L/h [74].

During another experiment, Papanikolaou et al. [63] examined the CA producing potential and the lipid accumulation in *Yarrowia lipolytica* strain after the inactivation of the 2-methyl-citrate dehydratase. The mutant *Y. lipolytica* JMY1203 was tested in nitrogen limited conditions and it produced up to 57.7 g/L of total citrate, with a glycerol to citrate yield of 0.92 g/g, and a productivity of 0.17 g/L/h. The fermentation process started from an initial substrate (crude glycerol or glucose) of 40 g/L [63].

Imandi et al. [16] evaluated the amount of citric acid production through a Doehlert experimental design, with the aid of the *Yarrowia lipolytica* microorganism, strain NCIM 3589. The maximum CA production was 77.399 g/L, resulting from 54.408 g/L of crude glycerol [16].

In another research paper, Papanikolaou et al. [75] suggest that *Y. lipolytica* strain LGAM S(7)1 gives a good biochemical response on raw glycerol as growth condition. At the end, relatively increased amounts of citric acid were produced, like 12 g/L, with a yield Y_Cit/Glol_ of 0.38 g/g and a specific CA production rate of 0.04 g/g/h. Growth and the parameters of citric acid production have been noticed to be comparable to those obtained from glucose, while glycerol intake was higher than intake of glucose, when *Y. lipolytica* is used [75].

Da Silva et al. [76] studied the bioconversion of crude glycerol resulted from biodiesel industry, into citric acid using *Y. lipolytica* IMUFRJ 50682 strain. They tested different initial concentrations of glycerol as nutrient substrate and added ammonium sulfate to the fermentation process. The substrate they used was obtained through the transesterification reaction of soybean oil with ethanol, and catalyzed by NaOH. Final products were analysed by high performance liquid chromatography (HPLC). At the end of the batch fermentation they observed that the citric acid production was about 12.94 g/L, in 160 h of fermentation of 45 g/L of glycerol. The use of ammonium sulfate during batch fermentation led to an increase of isocitric acid and a decrease of CA production, according to HPLC analysis. In the tests where ammonium sulfate was added (0.7 g/L) in order to determine the influence of nitrogen as a supplement source for citric acid production, the results pointed that the addition of ammonium sulfate to the culture medium leads to the metabolic path for the production of isocitric acid. In 93 h of fermentation it were obtained 16.79 g/L of isocitric acid and only 1.46 g/L citric acid when nitrogen was added. The authors concluded that a reduction in citric acid production is observed when nitrogen source is added to the fermentation process [76].

In the context of *Yarrowia*’s CA productivity, André et al. [77] tested the conversion potential of crude glycerol into citric acid by three different *Yarrowia lipolytica* strains (LFMB 19, LFMB 20 and ACA-YC 5033). In their submerged shake-flask experiments they used an initial concentration of 30 g/L of raw glycerol as a sole carbon substrate, leading to a satisfactory bacteria growth, a complete glycerol intake, and a good citric acid secretion. The authors suggest that for the strains *Y. lipolytica* LFMB 19 and *Y. lipolytica* LFMB 20, the principal metabolic product synthesized was mannitol (with a maximum concentration of 6.0 g/L, yield 0.20–0.26 g/g of CG consumed). These two strains recorded a low productivity of CA, such as 4.6 g/L with a yield of 0.25 g/g for LFMB 19 strain, and 3.2 g/L with a yield of 0.13 g/g for LFMB 20 strain. Opposite, the last named strain, *Y. lipolytica* ACA-YC 5033, registered simultaneously higher concentrations of both lipids and citric acid. It is noted that CA concentrations have increased with the increment of glycerol quantity, and the maximum total citric acid production was 50.1 g/L (yield 0.44 g/g of CG). From this report it can be observed that waste glycerol represents a proper carbon source for strains like *Y. lipolytica* [77].

*Yarrowia lipolytica* LFMB 20 growth was also tested by Papanikolaou et al. [13] on media containing high quantity of glycerol or high quantity of glucose. LFMB 20 produced citrate around 58 g/L in a culture with high-glucose intake, while on high-glycerol media around 42 g/L of citrate was produced, and about 18 g/L of mannitol. In batch cultures, when a mixture of glucose and industrial glycerol was used, citrate was obtained in a concentration of 53.4 g/L [13].

### *ASPERGILLUS’s* citric acid production

Even though such strain is well investigated for the production of citric acid, few reports are presented for its capacity to convert glycerol into CA. Generally, citric acid is created by submerged microbial fermentation on molasses using *Aspergillus niger* [2]. Even though *Aspergillus niger* is considered the main producer of CA by fermentation of different organic carbon sources, it seems that crude glycerol does not represent a good substrate for the production of citric acid. In this respect, the studies have been led on yeast strains (e.g. *Yarrowia lipolytica*) as an appropriate substitution. This can be explained by their resistance to high substrate concentrations and increased tolerance to impurities, allowing the use of low-quality substrates [8].

Xu et al. [78] investigated the effect of concentration and type of the carbon source on accumulation of citric acid in a batch fermenter induced by *Aspergillus niger*. In their report they presented a list of carbon sources (sucrose, maltose, fructose, glucose, glycerol, ethanol, succinate) tested for a high production of CA. The production of citric acid on glycerol was 17 mg/mL, starting from an initial carbon source concentration of 14%, while the results on maltose were obviously higher (49 mg/mL). Their work demonstrates that the concentration and the type of the carbohydrate source have a significant impact on the production of citric acid by *A. niger* [78] (Table 2).

**Table 2:** The concentrations and yields of CA from converted glycerol by *Yarrowia* and *Aspergillus* strains

Strain	Carbon source	CA concentration (g/L)	CA yield (g/g)	Productivity (g/L/h)	References
*Y. lipolytica* 1.31	Crude glycerol	124.5	0.62	0.88	[69]
	Pure/crude glycerol + glucose	71	0.45–0.46	0.57–0.69	[67]
	Crude glycerol	126–155	0.58–0.63	0.6–1.05	[68]
*Y. lipolytica* Wratislavia AWG7	Pure glycerol	51.2–86.5	0.59–0.67	0.78–1.59	[60]
	Pure/crude glycerol + glucose	76.6	0.46–0.48	0.76–0.81	[67]
	Crude glycerol	113.5–157.5	0.55–0.57	0.6–0.94	[68]
	Crude glycerol	154	0.78	1.05	[71]
*Y. lipolytica* K-1	Pure glycerol	75.7	0.40	0.81	[69]
	Pure/crude glycerol + glucose	36.8–53.3	0.27–0.3	0.51–0.64	[67]
*Y. lipolytica* A-101	Pure/crude glycerol + glucose	82.2	0.45–0.52	0.71–0.83	[67]
*Y. lipolytica* A-101-1.22	Crude glycerol	112	0.6	0.71	[70]
*Y. lipolytica* N15	Pure glycerol	98	0.70	1.14	[72]
	Crude glycerol	71	0.9	0.89	
*Y. lipolytica* NG40/UV7	Pure glycerol	115	0.64	0.80	[73].
	Crude glycerol	112	0.9	0.82	
	Crude glycerol + fatty acids	122.2	0.95	0.99	[9]
*Y. lipolytica* NRRL YB-423	Pure glycerol	21.6	–	0.94	[61]
*Y. lipolytica* ACADC 50109	Crude glycerol	62.5	0.56	0.1	[32]
	Glucose	50–55	0.83	0.6	[74]
	Pure glycerol	17.8	0.3	0.2	
*Y. lipolytica* JMY1203	Crude glycerol/glucose	57.7	0.92	0.17	[63]
*Y. lipolytica* NCIM 3589	Crude glycerol	77.39	–	–	[16]
*Y. lipolytica* LGAM S(7)1	Crude glycerol	12	0.38	0.4	[75]
*Y. lipolytica* IMUFRJ 50682	Crude glycerol	12.94	–	–	[76]
*Y. lipolytica* LFMB 19	Crude glycerol	4.6	0.25	–	[77]
*Y. lipolytica* LFMB 20	Crude glycerol	3.2	0.13	–	[77]
	Crude glycerol	42	0.39	–	[13]
	Glucose	58	0.55	–	
	Glucose + crude glycerol	53.4	0.41	–	
*Y. lipolytica* ACA-YC 5033	Crude glycerol	50.1	0.44	–	[77]
*Aspergillus niger*	Pure glycerol	17	–	–	[78]

## Conclusions and outlook

Crude glycerol is a biodegradable, safe, cheap, and reusable source of carbon, and presents a proper raw material for value-added compounds production. There is a considerable industrial interest in 1,3-propanediol and in citric acid production based on microbial fermentations, as it appears to be in competition with traditional technologies utilized to obtain such products. 1,3-propanediol is a very useful chemical compound with a major impact in the industry of biodegradable plastics, with a variety of applications. Citric acid is a widely used compound in the food industry because of its properties (flavor, antioxidant properties, taste, and low toxicity). It is considered that crude glycerol may become a valuable raw material for the production of different bio-chemical products, which will generate an important economic impact.

From this review it can be observed that microorganisms from *Enterobacteriaceae* family *(Klebsiella, Citrobacter, Lactobacillus*) and microorganisms from *Clostridiaceae* family (*Clostridium*) can be considered as good producers of 1,3-propanediol using crude glycerol as nutrient source (*K. pneumoniae* DSM 2026—61.1 g/L; *Cl. butyricum* F2b—93.7 g/L; *E. coli* K12—130 g/L; *L. diolivorans* DSM 14421—84.5 g/L) [6, 37, 43, 45]. For citric acid production, raised values were registered for *Yarrowia* yeast, and specifically *Yarrowia lipolytica* strain Wratislavia AWG7 gave a concentration of citric acid production of 157.5 g/L [68].

Waste glycerol from biodiesel industry can be considered an important raw material for future prospects, considering that it provides the carbon source for microbial growth. By recovering the waste glycerol from biofuels production and valuing it through bacterial fermentation in order to obtain value-added products, it has a positive economic and environmental impact.

## Abbreviations

1,3-PD: 1,3-propanediol
CA: citric acid
CG: crude glycerol
HLB: hydrophilic–lipophilic profile
PTT: polytrimethylene terephthalate
PET: polyethylene terephthalate
PBS: polybutylene terephthalate
D: dilution rate
OD: optical density
GC: gas chromatography
HPLC: high performance liquid chromatography
ESP: extraction screening program
TCA: tricarboxylic acid cycle
